# Cell2Cell: Explorative Cell Interaction Analysis in Multi-Volumetric Tissue Data

**DOI:** 10.1109/TVCG.2024.3456406

**Published:** 2024-12-03

**Authors:** Eric Mörth, Kevin Sidak, Zoltan Maliga, Torsten Möller, Nils Gehlenborg, Peter Sorger, Hanspeter Pfister, Johanna Beyer, Robert Krüger

**Affiliations:** Harvard Medical School.; Doctoral School of Computer Science at the University of Vienna.; Harvard Medical School.; Faculty of Computer Science and the Research Network Data Science at the University of Vienna.; Harvard Medical School.; Harvard Medical School.; Harvard University.; Harvard University.; New York University (NYU)

**Keywords:** Biomedical visualization, 3D multi-channel tissue data, Direct volume rendering, Quantitative analysis

## Abstract

We present *Cell2Cell,* a novel visual analytics approach for quantifying and visualizing networks of cell-cell interactions in three-dimensional (3D) multi-channel cancerous tissue data. By analyzing cellular interactions, biomedical experts can gain a more accurate understanding of the intricate relationships between cancer and immune cells. Recent methods have focused on inferring interaction based on the proximity of cells in low-resolution 2D multi-channel imaging data. By contrast, we analyze cell interactions by quantifying the presence and levels of specific proteins within a tissue sample (protein expressions) extracted from high-resolution 3D multi-channel volume data. Such analyses have a strong exploratory nature and require a tight integration of domain experts in the analysis loop to leverage their deep knowledge. We propose two complementary semi-automated approaches to cope with the increasing size and complexity of the data interactively: On the one hand, we interpret cell-to-cell interactions as edges in a cell graph and analyze the image signal (protein expressions) along those edges, using spatial as well as abstract visualizations. Complementary, we propose a cell-centered approach, enabling scientists to visually analyze polarized distributions of proteins in three dimensions, which also captures neighboring cells with biochemical and cell biological consequences. We evaluate our application in three case studies, where biologists and medical experts use *Cell2Cell* to investigate tumor micro-environments to identify and quantify T-cell activation in human tissue data. We confirmed that our tool can fully solve the use cases and enables a streamlined and detailed analysis of cell-cell interactions.

## 1 Introduction

*"Cells are the building blocks of life, from single-celled microbes through to multi-cellular organisms"* [39]. A better understanding of how cells interact with each other is essential to explain a multitude of biological processes, including disease mechanisms. Studying such interaction patterns has garnered significant interest in cancer research due to its potential to advance our understanding of immune response and identify drug development and therapy opportunities. Immunotherapy focuses on targeting immune checkpoint proteins that inhibit immune cells from identifying and killing cancer cells (see Fig. 2). Yet, therapy success highly depends on the patient’s immune response and type of cancer, and more research is needed to find and understand the role of checkpoint proteins in order to better target these interactions and improve therapies.

To capture cellular interactions, biological tissues are first imaged and digitized using high-throughput imaging technology. Highly multiplexed immunofluorescence (IF) imaging methods such as CyCIF [30] successively apply multiple fluorescent antibodies (also called markers) to the tissue. This makes protein expression visible, revealing information about the cells’ functional state and organization. Interaction between cells can then be statistically inferred based on the spatial position and proximity of cells [47]. To this date, most IF imaging data is two-dimensional, and while covering a large tissue area, the resolution per cell is low (a few pixels). Recent advances in IF microscopy [35], however, have now enabled the imaging of three-dimensional datasets with much higher (sub-cellular) resolution. Compared to a lower resolution and two-dimensional image, high-resolution 3D imaging can capture the spatial distribution of proteins, i.e., their location and intensity, around and between cells. Instead of inferring interaction based on spatial proximity, interactions become directly measurable by tracing protein expression values in 3D space, from *cell-to-cell* [35].

However, the lack of scalable and interpretable solutions renders interaction analysis in such 3D data infeasible. This is due to two challenges. Firstly, the analysis is exploratory, with many questions being ill-defined. Much of the knowledge needed to interpret protein distribution around and in between cells is exclusively internalized by pathologists and cell biologists. They have developed a deep visual understanding of human tissue and are skilled in identifying and verifying disease hallmarks by eye [24]. Leveraging this knowledge affords human-in-the-loop interfaces that visualize the data interactively in real-time. Secondly, there is a scalability challenge: Manual ( visual) analysis is infeasible for larger data and widespread use. Even a small number of cells can hold hundreds of interactions. For instance, 80 cells are involved in 200 interactions imaged with 32 protein markers. Going through all of these interactions manually and toggling several protein markers in an image analysis software is cumbersome and time-consuming. Thus, there is a pressing need for pathology and oncology to analyze and quantify cellular interaction more comprehensively and efficiently. Few software tools currently exist to assist in this task, and they rarely support combined visual and computational analysis.

**Fig. 1:**
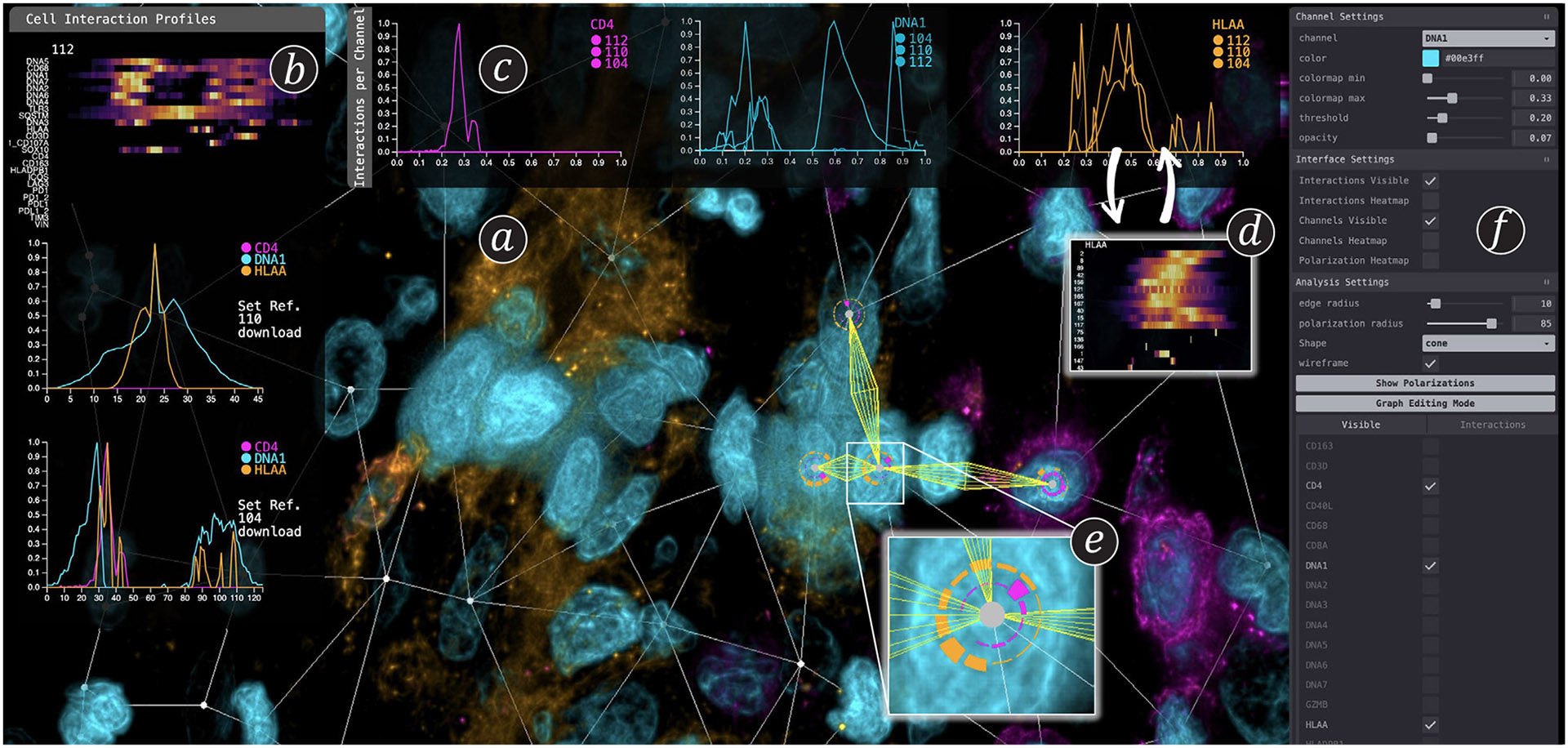
*Cell2Cell* is a web-based visual analytics system to analyze interactions of cells in 3D biological tissue imaging data. a) Multi-volume viewer using pseudo-colors. The embedded interaction graph displays cells (nodes) and their interactions (edges). b) Cell interaction profiles show the spatial intensity distribution of protein markers between cells. c) Multiple interactions can be compared channel by channel. d) Heatmaps (overview) and line charts (details) can be toggled on demand. e) Radial polarization charts enable cell-centric analysis. f) The side panel allows users to customize color settings and (de)activate channels.

**Fig. 2:**
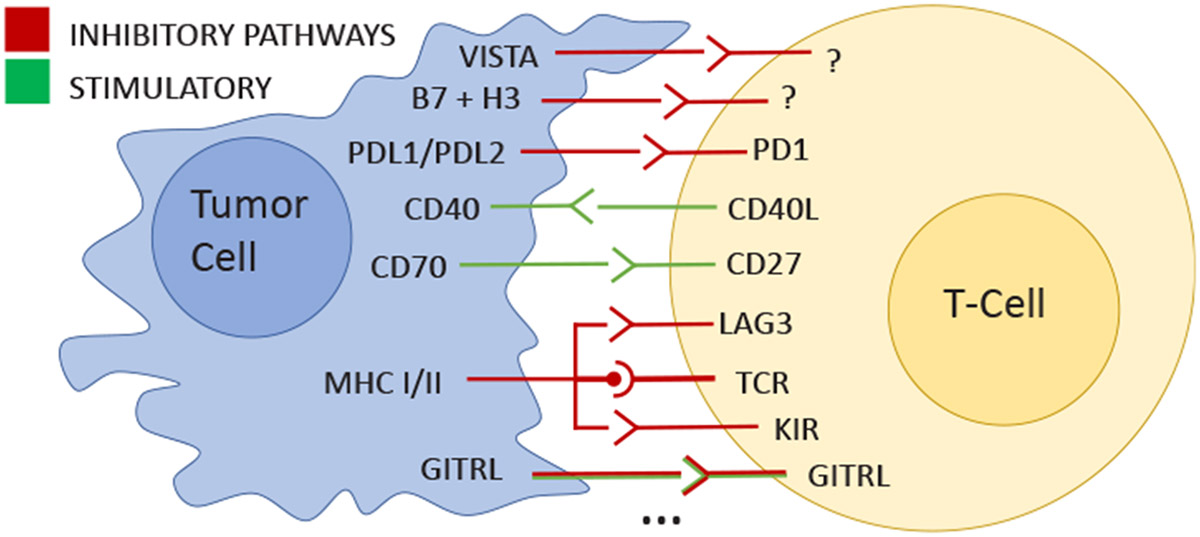
Immune checkpoint proteins (illustration based on Marin-Acevedo et al. [33]) can stimulate or inhibit immune cells from killing cancer cells. Understanding these interactions and when they occur enables the design of immunotherapies that block inhibitory checkpoints.

To address this gap, we collaborated closely with domain experts (medical doctors, systems immunologists, and computational biologists) to design a comprehensive visual and interactive system for parameterizing, analyzing, and quantifying cell-to-cell interactions in 3D CyCIF image data (see Fig. 1) based on identified domain goals and analysis tasks. Our work makes three main contributions:

**(1)** We designed a visual analysis **workflow for cell interaction analysis in multi-channel volume data,** which we implemented in *Cell2Cell.* **(2)** We present **a novel graph-based method for quantifying cell-to-cell interactions based on protein signal intensities** in a 3D setting. Our approach goes beyond the traditional neighborhood-based analysis, enabling domain experts to more accurately measure the sub-cellular distribution of molecular signals resulting from cellular interactions while incorporating expert knowledge to guide the process. In this graph, cells are conceptualized as nodes and interactions as edges connecting these nodes. We compute interaction profiles between two cells by tracing the surrounding protein expression along the connecting edge. We incorporated comparative views to assess the similarities and differences in interaction profiles across various samples, different cell types, or experimental conditions. **(3)** We propose **a cell-centered polarization analysis method** to study the circular distribution of protein markers around a cell. This allows analysts to gauge how (un)equally protein markers are distributed in the surrounding proximity, thereby indicating more nuanced behavior such as attractive and repelling interaction phenomena. This also allows the user to get an overview of potential interactions in the vicinity.

Finally, we assess our tool in three case studies involving domain experts: We demonstrate that earlier manual use cases [35] are reproducible in a fraction of the time required previously and show that *Cell2Cell* greatly aids analysis scalability and interpretability.

## 2 Related Work

In addition to dealing with multi-volume visualization aspects, we review previous work on cell interactions and spatial feature extraction. Since we are dealing with marker distributions, we also review related work on comparing and visualizing multivariate and radial distributions.

### Multi-Volume Visualization.

Multi-volumetric imaging datasets consist of multiple volumes that describe different properties of the same spatial object. In medical datasets, different 3D imaging modalities [27] or cyclic staining methods [30] result in different imaging channels (volumes) that are then registered and combined into a multi-volume dataset. In the biomedical domain, Agave [1], Napari [12], Voreen [15] and ViV [32] are among the most recent open-source tools that support interactive multi-volume rendering of various formats. Yet, the commercial sector tools like Imaris [2] and Arivis [20] are the most commonly used. While *Cell2Cell* contributes a similar multi-volume visualization implementation, its scientific novelty lies in the interactive analysis and comparison of cellular interaction and polarization patterns. We extend previous multi-volume visualizations to enable such analyses by embedding a cell interaction graph into the volume.

### Spatial Feature Extraction.

*Cell2Cell* uses spatial features of cells (i.e., the intensities of markers in and around a cell) to compute and display interaction profiles. The state-of-the-art approach for quantifying cellular features in IF data is to compose a single-cell table that comprises one row per cell, with mean intensity values for each imaging channel [41]. This originates from low-resolution 2D imaging where a cell comprises a few pixels only, so information loss is minimal. For higher-resolution 2D and 3D data, many approaches use shape descriptors [28] that require a prior cell segmentation step. Ternes et al. [44] employ variational auto-encoders (VAEs) to extract transform-invariant biologically meaningful features from two-dimensional imaging data. Johnson et al. [25] employ VAEs to learn cell and nuclear morphology shapes and variations from multiplex 3D data. Their approach, however, requires segmented cells as input and many examples in a curated training set. They then create a catalog of cell types and states. In contrast, our approach does not need full cell segmentation and focuses on interactions between pairs of cells. While auto-encoders effectively capture features in multi-volume data, they are hard to interpret. Instead, our expert’s domain conventions are to compute a 1D spatial intensity profile [35] that accumulates and aggregates a defined space between cells, enabling interpretable analysis.

### Visual Analysis of Cell Interactions.

Histocat [40], Imacyte [43], and Visinity [47] are visual interfaces to analyze cellular interaction patterns in tissue imaging data of increasing scale. These approaches compute interactions based on the distance between segmented cells. Histocat and Visinity display interaction in image space by colored cell outline overlays and outside of the image by node-link diagrams and other plots. Imacyte proposes a glyph-based encoding of cellular neighborhoods, including frequency, variation, and significance. All these visualizations focus on inferred interaction networks. By contrast, with *Cell2Cell,* we visually display and analyze the real imaging signal between cells. Like our approach, Barrio [45] investigates 3D morphology and interactions between cell organelles in single-channel electron microscopy data. However, Barrio focuses on visual comparison instead of ranking and grouping patterns computationally. Further, the approach does not propose a solution for multi-channel data.

### Visualization of Spherical and Radial Distributions.

Several works have addressed the visual display of spherical distributions. Grundy et al. and Wilson et al. propose spherical histograms [21] and orientation spheres [48], similar to Fritz et al. [16]. Our requirements are different because we focus on polarization and repelling of signals with respect to the interaction space between cells only. This allows us to use a more abstract visual encoding that displays such distributions on a simpler and more pre-attentive radial chart instead of a sphere. Draper et al. [14] survey radial charts. Our approach is most similar to stacked radial area charts and star plots, as used by Mörth et al. [34], but we use multiple circles to encode the multivariate data features to ease comparison. We draw these charts on top of the nodes in a network. Nobre et al. [36] summarize work on visualizing such multivariate networks, and Gehlenborg et al. specifically review interaction encodings in the biology domain [18]. Most network visualizations are, however, non-spatial. More similar to our encoding is work in the geographical domain [29,51], where temporal or directional information is encoded in nodes of a geospatial network.

### Visual Comparison of Multivariate Distributions.

Gleicher et al. [19] propose a taxonomy for visual comparison with three fundamental visual designs: juxtaposition, superposition, and explicit encoding. Our approach leverages superposition and juxtaposition to compare cell interaction profiles. Both Gleicher [19] and Yi et al. [50] propose interaction strategies for such visual comparison techniques, including interactive highlighting (Yi’s connect category) and reordering/re-arranging (reconfiguring) objects. We use both techniques to emphasize similar marker profiles across pairs of cells (1) and to compare marker profiles to a selected reference interaction (2). However, *Cell2Cell* is specific to comparing multivariate line (marker) profiles. Javed et al. [23] study graphical perception of multiple time series visualized as line graphs. They found that visualizations using superposition and explicit encoding are usually better for comparing short-line graphs. On the other hand, juxtaposition approaches are better suited for comparing long time series. As we compare within and across pairs of cells, we use both techniques, following the concept by Yi et al. [50] to combine multiple visual comparison techniques to solve a specific problem.

### In a nutshell,

there are a small number of web-based multi-volume visualization tools, of which even fewer handle single-cell tissue data. Moreover, most existing cell-cell interaction analysis approaches are based on proximity assumptions in low-res 2D data. Those that concern 3D data do not offer explorative visual environments and often require segmented data. By contrast, *Cell2Cell* supports protein (intensity)-based interaction analysis for unsegmented high-res 3D data.

## 3 Background - 3D Multichannel Tissue Data

Cyclic Immunofluorescence (CyCIF) [30] microscopy imaging measures the distribution of proteins in biological tissue. By successively staining the tissue with multiple fluorescent antibodies (also called markers), this method outputs a set of 2D images (image channels), where each channel depicts the intensity of a certain protein marker.

**Fig. 3:**
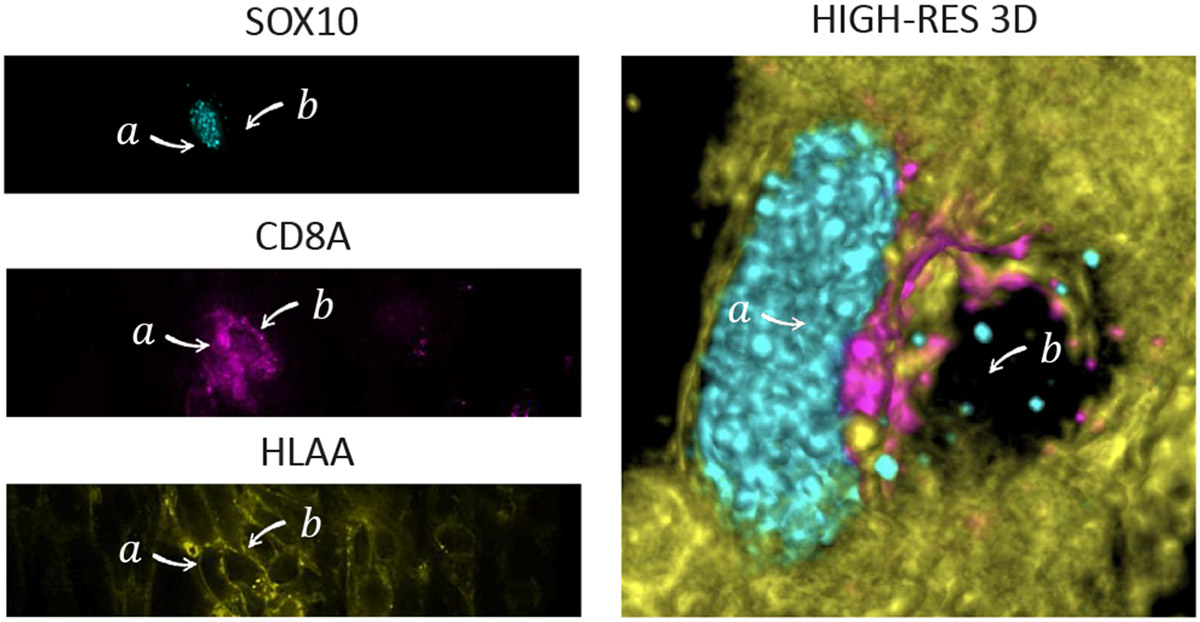
Expression pattern of 3 representative proteins (i.e., channels) out of 28 channels in a CyCIF dataset. Staining of SOX10 (cyan) labels the nuclei of tumor cells, and CD8A (magenta) labels the plasma membranes of cytotoxic T lymphocytes associated with tumor killing. HLAA (yellow) labels the plasma membranes of all cells. The 3D mult-volume rendering provides a detailed spatial overview of tumor cell *a* and a T-cell *b.*

Each channel (see Fig. 3 showing 3 such channels) provides information on the cell lineage (tumor or specific functional class of immune cell). Furthermore, the spatial arrangement of markers within the cell generates testable hypotheses of (1) cell functional state (2) the role of a marker in tumor biology. In the example of melanoma, high SOX10 levels co-staining with DNA identify tumor cells, whereas CD3D forming a circle around a cell indicates the boundaries of a cell with the potential for tumor-cell killing.

A CyCIF dataset can consist of up to 60 channels, with each individual image having 10^9^ or more pixels per channel and containing millions of cells. Fig. 3 shows three different channels in a tissue sample. Recently, confocal immunofluorescence microscopy has been used to image optical sections in higher resolution and at different depth levels. These sections can be registered to reconstruct a 3D volume of the tissue. The novel addition of higher resolution 3D volumetric data (see Fig. 3, right) has the potential for novel insights, as many structures that appear disconnected in sections are expected to be part of a single large structure in 3D [31]. This is expected to benefit spatial analysis questions, e.g., concerning tissue morphology, cell shapes, and spatial cell neighborhood and correlations.

## 4 Goals and Tasks

Following the design study methodology by Sedlmair et al. [42], we have identified the following domain goals and analysis tasks for cell-cell interaction analysis. We defined the goals with two domain scientists, one holding a Ph.D. in Biophysics and currently studying the immune responses to cancer in a real-world context. The second expert is an Associate Professor of Pathology who practices neuro-pathology focusing on the molecular pathology of brain tumors. The goals were extracted and refined through regular monthly meetings and communication with the domain experts in an iterative process over 17 months. We reconstructed previously conducted analyses and explored potential new analysis methods for current and upcoming CyCIF datasets. This collaborative and iterative approach allowed us to effectively identify and refine the domain goals and tasks that *Cell2Cell* aims to address.

### 4.1 Domain Goals

Our collaborator’s objective is to analyze and parameterize interactions between neighboring cells in 3D images. They want to analyze interactions between cells at a higher resolution than previous phenotyping approaches that used spatial statistics of single-cell neighborhoods but ignored the detailed sub-cellular distribution of proteins [35,47]. Our collaborators are interested in a) discovering novel functionally significant cell-cell interactions and b) screening for instances of known interactions in the data. Specifically, their goals are:

#### G1 - Data Quality Assurance.

The domain scientists are greatly interested in assessing the quality of their data. Every step in the processing pipeline can lead to potential errors in the downstream analysis. Analyzing cellular interactions aims to ensure that the data loaded into the analysis tool (i.e., imaging volumes and labeled cell center positions) is correct.

#### G2 - Understanding Interactions between Cells.

Our domain experts want to screen for cell-cell interaction patterns consistent with cancer progression and response to drug therapy. Therefore, they need to rapidly quantify and parameterize the distribution of proteins at cell-cell boundaries. Of specific interest are so-called immune checkpoint proteins that can inhibit or stimulate immune cells from detecting and killing cancer cells. Currently, the process of quantifying cell interactions in multi-channel and high-res 3D data is labor-intensive, requiring experts to manually examine the data and overlay simple geometric shapes on portions of the images to generate histograms [35]. By addressing the limitations of this manual approach, their goal is to more efficiently and accurately study and understand the intricate relationships between cells in a 3D context, ultimately contributing to advancements in cancer and immunology research.

#### G3 - Understanding Protein Polarization in Cells.

In addition to cell-to-cell interactions, our domain scientists also want to examine how protein markers are distributed within a cell (cell-intrinsic **polarization profiles**). This has several reasons. Firstly, cells might have multiple interactions, so looking at them from a cell-centric perspective, with the spatially proximate tissue context available, gives a more holistic view. Secondly, immune reactions in an early stage usually express proteins within the cell before breaking through the membrane. Thus, they may not be present at the cell boundaries yet. Thirdly, it can aid verification. A protein that is evenly present in all directions instead of exclusively targeting a specific neighboring cell may falsify an interaction hypothesis. An example derived through previous manual computation involved productive (killing) recognition of tumors by immune cells: A tumor-killing marker (CD8A) was directed to the tumor-immune cell interaction (immune synapse). In contrast, inhibitory proteins (LAG3 and TIM3) are directed away from this structure [35].

### 4.2 Taxonomy

The data characteristics and domain goal descriptions lead to the following taxonomy: An **Image Channel** is one of up to 60 volumes in a tissue dataset. Each image channel holds the intensity distribution of a protein across the tissue. A **Cell** is a three-dimensional region in the tissue volume with protein expressions across channels. The **Cell-Graph** is an abstract concept of tissue organization to aid cell-cell interaction analysis. Cells are reflected as nodes (with center point [x,y,z]), and edges represent the shortest paths between cells. An **Interaction Profile** captures and quantifies the protein distribution between two cells in a given distance around their connecting edge (shortest path). A **Polarization Profile** captures and quantifies the spherical protein distributions in a given distance around a cell center (cell-centric).

### 4.3 Analysis Tasks

Given the goals and taxonomy, we derived the following analysis tasks.

#### T1 - Explore Multivolume Data.

Before the analysis, domain scientists need to gain a visual overview of the spatial organization of cells and their protein expressions in the imaging data (**G1-3**). The experts want to be able to inspect 3-4 channels at most together but in flexible combinations. A focus is exploring protein signals in and around cells from adjustable viewing directions.

#### T2 - Proofread Cell Centers and Interaction.

To analyze the interaction between cells, the position of cells needs to be computationally captured (labeled). This is typically done in a preprocessing step. Scientists need to verify the position of labeled cell centers within the underlying imaging data and adjust center positions if needed (**G1**).

#### T3 - Analyze Interactions of Interest.

Analyzing the interaction between cells requires the ability to spatially find an interaction of interest in the image and select the relevant combination of protein channels for the task. Subsequently, the scientists want to look at the selected interaction profile in detail and trace and examine the protein distributions between the two cells in focus (**G2**).

#### T4 - Compare Cell-Cell Interactions.

Similar interaction profiles may indicate a relevant biological phenomenon. To find (dis-)similar interactions to a given interaction, the domain scientists thus need the ability to compare interaction profiles (**G2**). This includes sorting and ranking interactions based on similarity and being able to organize (group) them to identify repetitive patterns as well as outliers.

#### T5 - Analyze Cell Polarization.

Complementary to analyzing cell interaction, experts want to explore the spherical distribution and polarity of one or multiple markers in and around cells from a cell-centric point of view (**G3**). A cell’s polarization needs to be explorable in its spatial context, i.e., how the surrounding contributes to the distribution.

#### T6 - Export Findings.

To continue downstream analysis in other software, the domain scientists must export details on identified interactions and cell polarization data.

## 5 *Cell2Cell* Design

We used the identified goals and tasks to inform the design of *Cell2Cell.* The main analysis concept is to leverage (scan) directly the protein signals around and in between cells in the 3D data as opposed to any purely distance-based inference of interaction.

### Graph-Based Cell Interaction Analysis.

In the first step, we decided to abstract the representation of our problem domain into a graph/network context. A graph offers an intuitive and efficient representation of a network of interacting cells. In this context, cells can be conceptualized as *nodes*, with their intricate protein interactions as the connecting *edges.* This abstraction simplifies the complex nature of cellular interactions and provides a structured framework for analyzing and visualizing these interactions.

By basing *Cell2Cell* on the concept of a *cell graph* (Sec. 6). we can support an interaction-centric and cell-centric analysis workflow, focusing on edges and nodes. To further simplify our representation, we approximate cells as spherical shapes. This assumption holds for most of the cells in our data, but our representation could be extended to ellipsoids or more complex shapes in the future. A spherical approximation allows us to rely only on labeled cell center positions for our workflow, circumventing the need for complex cell segmentation.

### Preprocessing.

*Cell2Cell* requires multi-channel volume data of tissue samples (e.g., CyCIF data) and labeled cell center positions. Full cell segmentation is still challenging and time-consuming as ground truth data and pre-trained models are rare, especially for data from novel imaging technologies. Our approach instead relies on labeled cell centers (x,y,z positions in the volume). This is quicker since it does not require the extraction of complete and accurate cell boundaries. Currently, our collaborators use Ilastik [8] for annotating cell centers, which takes half an hour for a typical tissue sample (1,024 x 1,024 x 55, around 80 cells). However, depending on the preference of the domain scientists, more automatic methods could be used.

### Workflow.

**Fig. 5:**
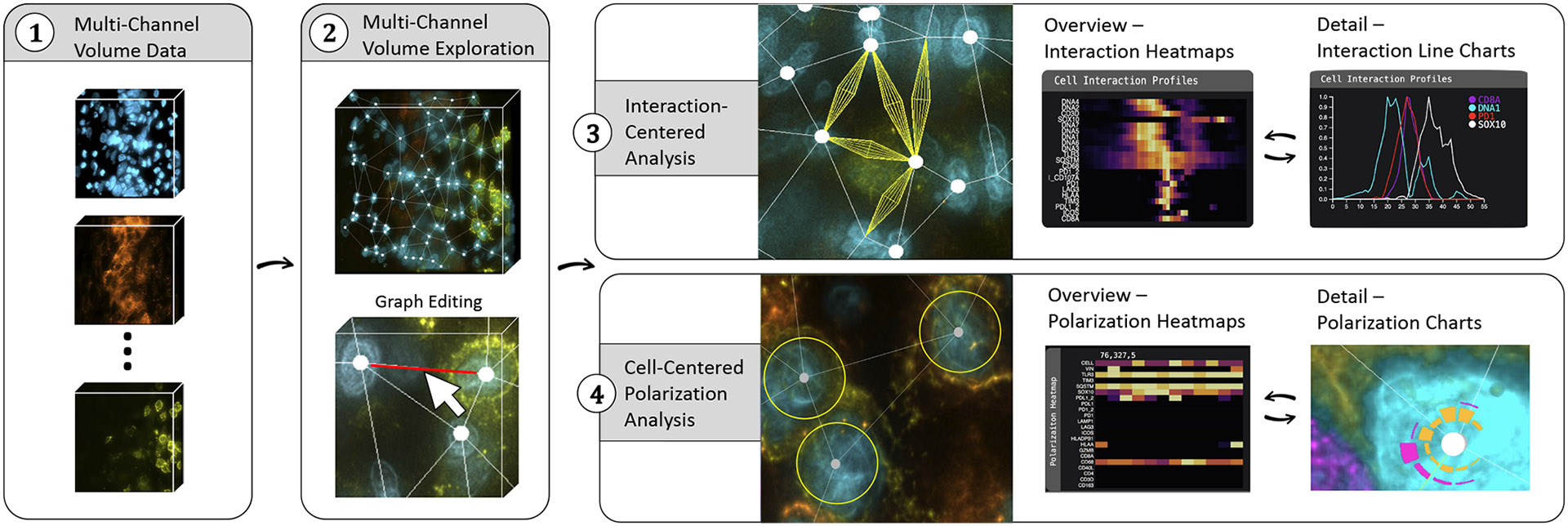
*Cell2Cell*’s workflow starts with visual exploration (2) of the multi-volumetric imaging data (1) and embedded interaction graph. Users can then perform interaction-centered analysis (3) by looking at single interactions or comparing multiple interactions. Alternatively, users can perform a cell-centric analysis (4), focusing on polarization patterns of protein markers around a cell. Both interaction and polarization can be analyzed in linked heatmaps (overview) and line and polarization charts (detail).

Fig. 5 depicts the workflow in *Cell2Cell.* After loading in multi-channel volume data, users start with a high-level *visual exploration* **(T1)**, leveraging multi-volume rendering at interactive rates. *Cell2Cell* enables scientists to analyze cellular interactions based on our concept of a cell graph (Sec. 6). Users can *proofread* **(T2)** the cell graph to verify the positioning of nodes and edges and, if necessary, add, delete, or adjust them. Next, users can perform two complementary analysis approaches: *interaction-centered analysis* **(T3)**), where the focus is on the edges of our cell graph; and *cell-centered polarization analysis* **(T5)**, where the focus is on the nodes of our cell graph. For both modes, *Cell2Cell* offers visual tools for exploring and comparing the computed profiles **(T3-5)**. Finally, findings can be *exported* for downstream analysis and presentation purposes **(T6)**.

## 6 Cell Interaction Graph

*Cell2Cell*’s workflow is based on a cell graph representing cells and their interactions as nodes and edges. We describe the cell graph computation in Sec. 6.1 and optional graph editing features in Sec. 6.2.

### 6.1 Graph Computation

Based on a provided cell center annotation, *Cell2Cell* generates a cell graph *G* = (*V, E*) where each node in *V* represents a cell center and edges *E* represent potential interactions between cells. To construct the graph, we employ a 3D Delaunay triangulation, as it captures the natural connectivity of data points in space, making it more representative of biological cell networks [11, 13, 37]. It also aligns with the biological assumption of our domain experts: Two cells (nodes) *a* and *b* are likely to interact with one another only if there is no other closer cell *c* directly in between. Currently, our volume is thin in the z-dimension, which leads to almost planar graphs. To further trim the number of initial interactions (edges), we compute the Gabriel graph [17]. Its disc-area criterion ensures that no cell *c* is close to an interaction between two other cells *a* and *b*, i.e., *c* does not lie within the disk enclosing the edge *{a, b}*. We heuristically found this to be a good approximation for biologically relevant interactions since cells only interact with each other if there is no other cell between them.

### 6.2 Interactive Graph Editing

Since the imported cell centers might be inaccurate or incomplete, we also provide means to edit cell center positions and the resulting cell graph **(T2)**. *Cell2Cell* offers a slice view feature that allows domain experts to precisely place and adjust the position of nodes (i.e., cell centers). The primary objective of this feature is not to replace specialized segmentation or annotation tools but to provide a means for correcting node positions as needed. We communicate each added or removed node to the backend and automatically recalculate the cell graph to incorporate the user’s changes. This is done at run time and typically takes a few seconds. Adding or removing edges is conceptually similar and can be supported by the slice view.

## 7 Interaction-Centered Analysis

*Cell2Cell*’s interaction analysis **(see Task T3)** is based on the concept of *interaction profiles.* We utilize our cell graph structure to compute interaction profiles between any two cells. We traverse the volume along the graph’s edges and automatically compute the change of marker intensities along an edge, resulting in an interaction profile for each edge (see Sec. 7.1). To visualize the computed interaction profiles, *Cell2Cell* has to handle two separate scalability challenges: (i) interaction profiles contain data from *many data channels,* and (ii) users might want to look at and compare *many cell interactions*. In Sec. 7.2, we describe our overview-and-detail approach for analyzing single interactions with many channels. Sec. 7.3 describes how we support comparing many cell interactions.

### 7.1 Cell Interaction Profile Computation

**Fig. 6:**
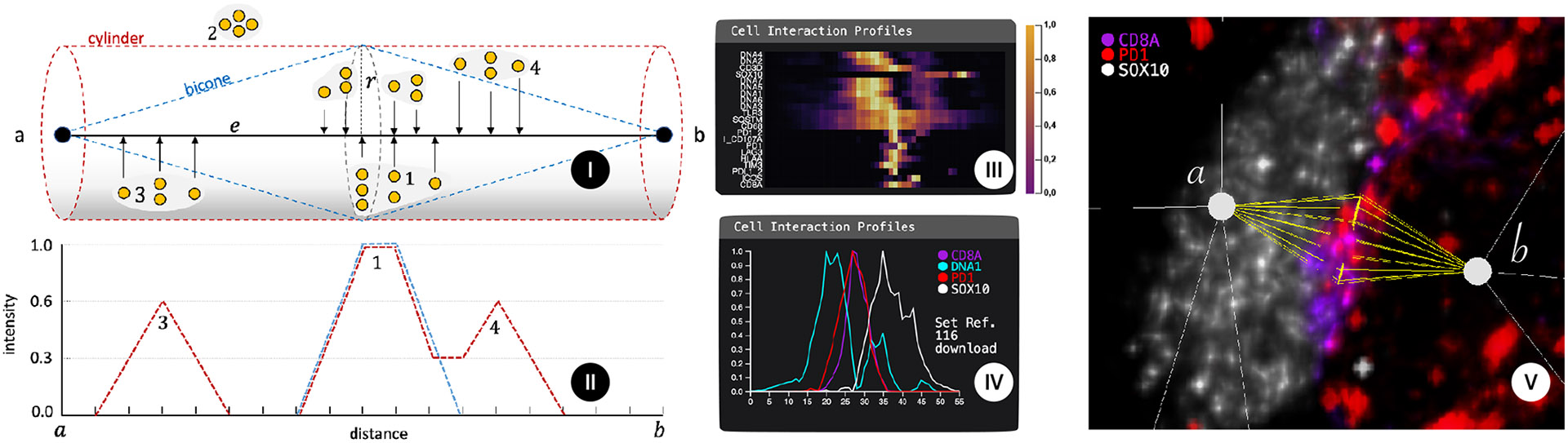
**(I)** Cell interaction profile calculation around one edge with one channel (yellow). Bicone (blue) or cylinder (red) with a base radius (*r*) are positioned at the endpoints of the edge (*e*), which connects two nodes (*a*, *b*). Voxels inside the shapes (e.g., 1) are projected onto the edge (*e*), and outside voxels (e.g., 2) are ignored. The mean of the projected values determines the intensity at the corresponding location on the edge, for each channel individually. **(II)** The line chart shows the accumulated intensities at each point along *e* between nodes (*a, b*). Note the difference between bicone and cylinder. **(III)** Heatmap encoding of all markers with expression along the *a-b* (tumor cell—T-cell) axis. Rows represent channels, the x-axis represents the position along the interaction edge. Color encodes marker intensity, channels are ordered by similarity. SOX10 is expressed in a tumor cell (a), and different membrane markers (e.g., DNA7) are localized at the midpoint between cells. **(IV and V)** Interaction profile between tumor cell (*a*) and T-cell (*b*) showing the distribution of 4 markers. Notably, the maximum intensities of the membrane proteins CD8A (magenta) and PD1 (red) indicate an immunological response between tumor (SOX10, white) and immune cell (DNA1, cyan, only shown in the interaction profile).

To quantify the interaction between two nodes (cells) *N*_1_, *N*_2_, we accumulate marker intensities surrounding the respective edge *e* in the graph into an interaction profile (see Fig. 6). More precisely, we integrate the intensity of each voxel within a specified radius (depicted as *r*) orthogonal to the edge *e* onto the closest point on the edge. We then calculate the mean intensity for the respective edge position. This results in a one-dimensional distribution (interaction profile) for each image channel (see Fig. 6 II). The values are furthermore normalized between 0 and 1 based on the overall value distribution of the given interaction profile. We support two different geometric shapes to define this area: cylinders and bicones. Bicones reflect the idea of capturing spherical sectors emanating from cell centers along a cell-cell interaction vector. With a growing diameter towards the center, bicones consider the spherical shape of cells and focus on the cell’s surfaces and area of contact. Cylinders, by contrast, have been used in prior work for their simplicity [35] and capture the same interaction with a fixed spatial cross-section. *Cell2Cell* allows experts to choose between the two shapes and to interactively set the aforementioned radius based on domain knowledge and use case.

### 7.2 Single Interaction Analysis

We visually embed the computed cell graph in the volume rendering to enable users to explore cellular interaction directly in their spatial context (see Fig. 1). Users can directly select edges of interest in the volume view and then follow an *overview and detail* approach to analyze the selected interaction.

#### 7.2.1 Overview: Interaction Heatmaps

As a first step, experts want to find the channels of interest for a particular interaction (e.g., channels that show an interesting pattern in their interaction profile). Since typical datasets contain 20 or more channels, we chose a compact heatmap visualization (Fig. 6 III) to give the domain experts a first glance at the role of different channels in an interaction. Each row in the heatmap represents an image channel. The columns represent the interaction length (i.e., voxels along the corresponding edge of the cell graph). As shown in Fig. 6 III, only some channels are typically visibly active in a selected interaction. The expert can then choose the interesting channels for further investigation.

#### 7.2.2 Detail: Interaction Profile Line Charts

Once users have identified channels of interest in an interaction, they can analyze them in more detail. The *interaction profile line chart* (see Fig. 6 IV) depicts each selected channel as a separate line, with the x-axis representing the interaction edge and the y-axis representing the channel’s intensity at that position of the interaction edge. The line chart is connected to the volume view via brushing and linking. As edges in the cell graph are undirected, users can hover over a position in the line chart to highlight the corresponding spatial position in the volume view (see Fig. 6 V). Line charts are a fitting visualization since we reduce the protein marker distribution from 3D to a one-dimensional distribution along an edge. Further, they offer several advantages:

##### Clarity and simplicity.

Line charts are easy to understand, allowing researchers to grasp the trends and changes across the edge quickly.

##### Comparison of multiple channels.

Line charts can visualize multiple channels within one plot. This enables the user to compare channels and see patterns and correlations between different channels.

##### Scalability.

Line charts are well-suited for handling a varying number of channels. Despite the limitations of finite color encodings, the ability to highlight channels when hovering over them ensures clear perception and comprehension of the displayed data. Furthermore, line charts are a good option for a small multiples display when comparing several interactions, as described in Sec. 7.3.

### 7.3 Cell Interaction Comparison

Building on well-established principles from the field of visual comparison [19], our tool incorporates several views to analyze and compare **(T4)** several interactions simultaneously. To keep the user experience consistent, we use the same visual encodings as in the single interaction analysis (i.e., line charts and heatmaps). However, we use small multiples and the concepts of juxtaposition and superposition to compare interactions efficiently. We further automatically compute an edge similarity score to group similar edges together. We focus on two main aspects: comparing interactions edge-by-edge and channel-by-channel.

#### 7.3.1 Similarity Computation

*Cell2Cell* eases comparing interaction profiles by automatically ordering them by similarity. Integrated into heatmaps and line charts, this feature allows users to sort profiles based on a selected reference profile. To account for differences in the lengths of interaction profiles, we first normalize their length based on the length of the selected (primary) edge using linear interpolation. Interactions are then compared using the Wasserstein distance [26] (Eq. 1). The metric aligns with our design requirements that two interaction profiles are most similar if one can be transformed into the other with minimal costs [38]. The Wasserstein distance finds similarity even in phase-shifted signals. It also has advantages over sequence distance measures such as Dynamic Time Warping [9] because it satisfies the triangle inequality.

(1) $$W_{p}(P,Q)={\left(\frac{1}{n}\sum_{i=1}^{n}{\left\|X_{i}-Y_{i}\right\|}^{p}\right)}^{\frac{1}{p}}$$

P is an edge of length n with values $X_{1},\ldots X_{n}$ and Q depicts a second edge of length n with values $Y_{1},\ldots Y_{n}$. Our moment p is 1, matching the 1D distribution. The distance between each channel is calculated, with the total distance being the sum of these individual distances.

#### 7.3.2 Small Multiples Display

*Cell2Cell* supports two types of small multiple displays for edge-by-edge and channel-by-channel comparisons, respectively. Both types work conceptually similarly for heatmaps and line charts alike.

##### Edge-by-edge Comparison.

Once users select several edges of interest, we display them as small multiples in a vertically arranged list, either as heatmaps or line charts. The visualizations are identical to the single-edge analysis case, where each individual visualization represents one interaction (see Fig. 1, vertical line chart panel). Following the overview and detail metaphor, heatmaps can quickly identify interesting channels, which can be analyzed in more detail in the line charts. Juxtaposing the individual views limits complexity and avoids overplotting.

##### Channel-by-channel Comparison.

To compare the channels of multiple cell-to-cell interactions in more detail, we arrange small multiples in a horizontal list, where each visualization focuses on one channel (see Fig. 1, horizontal line chart panel). That means we show one heatmap per channel, where each row represents an interaction and the intensity of that single channel along the edge of the interaction. For line charts, each chart represents one channel, with interactions being superimposed as individual lines. As before, heatmaps are used for an overview of many interactions, while line charts are used to examine selected interactions in detail. All views allow brushing and linking to highlight positions and features in the volume view for spatial context.

## 8 Cell-Centered Polarization Analysis

**Fig. 7:**
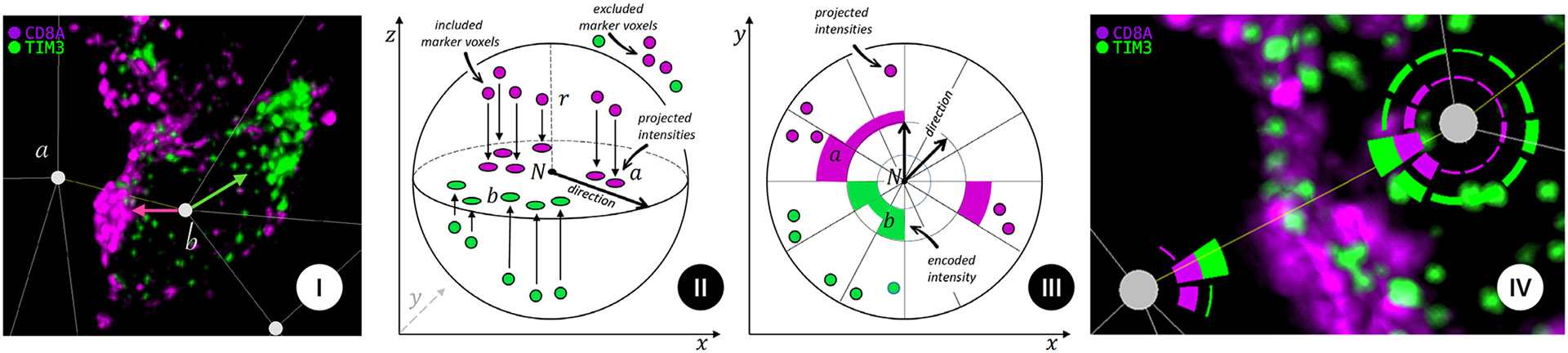
**(I)** Cell polarity in a tissue context. Cell interaction network centered on tumor cell (*a*) and immune cell (*b*) reveals polarization (arrows) of CD8A (magenta) to tumor cell (*a*) but TIM3 (green) away from all recorded cell-cell interactions. **(II and III)** Calculating marker polarization. **(II)** We compute cell polarization in a sphere with the radius (*r*) surrounding the cell (node) *N*. Only voxels inside the radius are included in the calculation of the cell polarization. We orthogonally project marker values onto a 2D plane to create a 2D map of the cell’s polarization. **(III)** Top-down view (*x,y*) of the projected intensities. Our radial visualization displays marker intensities as concentric rings (*a,b*), divided into smaller circle segments indicating the direction of the respective marker intensity, encoded by width. **(IV)** Polarization of two cells on the example of two channels. Channels are displayed as concentric circles. The direction and strength of protein signals from these channels are encoded by the thickness of the 12 circle segments. We see a polarization towards each other, a strong indication of Cell2Cell interaction.

In addition to the interaction-focused analysis, *Cell2Cell* also enables scientists to perform a cell-centric analysis (T5). A spherical model of a cell-centered on the nucleus permits either spherical (3-D) or polar (2-D) parameterization of marker distribution. In general, markers in the cell’s center are labeled nuclear. In contrast, markers at the plasma membrane (i.e., at cell-cell contacts) can be evenly distributed around a cell or concentrated (polarized) towards or away from a neighboring cell. For example, in Figure 7 I, CD8A is highly polarized toward tumor cell a with TIM3 being polarized to the top left of cell b.

### 8.1 Cell Polarization Computation

To compute the polarization of cells, we analyze the marker distribution within a certain distance around the cell core. We approximate the cell’s shape as a sphere, which allows us to analyze all voxels within a radius *r* around the cell’s center. Next, we project marker intensities inside the sphere onto a 2D plane centered at the cells’ center and accumulate marker intensities for every pixel on that plane (see Fig. 7 II and III). We then perform an orthogonal projection onto the 2D viewing plane of the volume view. This projection approximates the actual 3D spherical distribution of a marker. However, it allows us to use a visually simpler 2D encoding to display the polarization data (see Sec. 8.2) that is always aligned with the current view direction in the volume view. Whenever the view direction changes, we recompute the polarization based on the new viewing plane orientation.

### 8.2 Cell Polarization Visualization

To visualize cell polarization **(T5)** we again follow an overview-detail metaphor. We use a heatmap-inspired display to show the polarization of a cell for all channels, and a circular polarization chart embedded into the volume to show cell polarization for selected channels of interest.

#### Circular Polarization Chart.

Since cell polarization is directly related to a cell’s spatial neighborhood (in which direction neighboring cells are located), we display polarization information directly in the volume view around the cell. For each node, the radial cell polarization visualization shows a projection of the spatial distribution of protein markers around that cell onto a 2d disc. If the volume is rotated, we recompute the projection and update the radial polarization charts. Each polarization chart can show the spatial distribution of a selected number of channels. Channels are represented as concentric circles. Each circle is divided into 12 sections, similar to a clock face. The width of each circle segment encodes the intensity of the corresponding channel in that area, similar to a radial bar chart (see Fig. 7 III). Thus, each channel’s polarization is displayed in its own orbit surrounding the cell center (see Fig. 7 IV). After consulting with the domain scientists, we chose to accept the drawback of the areal increase towards the outer orbits due to the more significant advantages of the design: the radial encoding allows us to intuitively understand a cell’s direction of polarization.

#### Overview and Detail

**Fig. 8:**
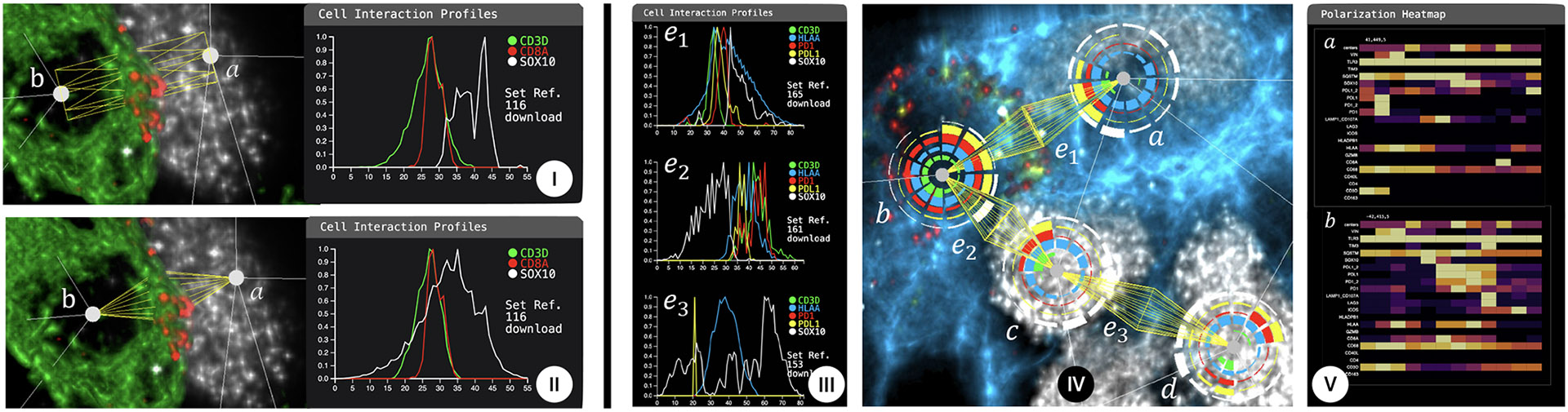
Case Study 1 : **(I, II)** Different geometries to parameterize cell-cell interactions. Interaction of tumor cell (*a*) and cytotoxic T lymphocytes (*b*) using cylindrical and conical integration. Channels rendered and interactions visualized: CD3D (green), CD8A (red), and SOX10 (white). **(I)** A cylinder is used to capture the interaction profile. **(II)** A bicone shape is used. — Case Study 2: **(III, IV and V)** Quantifying immune suppression of T-cell (*b*) by tumor cell (*a*). PD1 (red) and binding partner PDL1 (yellow) co-localize between cells *a* and *b,* as quantified along edge *e*_1_, *e*_2_. A similar co-distribution does not occur in *e*_3_ between tumor cells *c* and *d.* Polarization heatmaps (V) give an overview of all markers for cells *b* and *a.*

With the above-described circular charts, scientists can see a cell’s polarization in combination with the spatial context of a cell to understand the biological underpinning. However, the circular charts work best for showing 1-4 channels. We propose a heatmap-inspired encoding to get an overview of a cell’s polarization for all its channels. Each channel is represented as a different row in the heatmap. To compute a row, we conceptually *unroll* the channel’s circle of the radial chart into a straight line and convert the height of bars to a color scale. This allows us to represent the polarization information of all channels around a cell in a compact view. A limitation of this approach is that we cannot intuitively depict the actual direction of polarization since the circular chart is unrolled. However, it is easy to spot polarization patterns (i.e., polarized vs. evenly distributed) and to identify channels of interest (see Fig. 8, V) that can be explored in more detail in the circular polarization chart (Fig. 8, IV).

## 9 Implementation

*Cell2Cell* is a web-based client-server system with a RESTful interface. Users can access and interact with the tool in a web browser. We plan an open-source release upon publication. The **backend** of our application is implemented in Python, utilizing the Flask web framework [5] to handle server-side operations. For data processing and analysis, we use NumPy [3] and scikit-image [46]. The **frontend** of our application is developed using TypeScript. To render the 3D multi-channel volume data and cell graphs, we use the Three.JS library [4]. For generating the various plots and charts required in our interaction and polarization analyses, we use D3.js [10]. Our WebGL-based direct volume renderer can render up to six channels simultaneously. We limit the number of channels to prevent visual clutter, overplotting, and interactivity.

## 10 Evaluation

We evaluate *Cell2Cell* in three use cases and interviews with two domain experts. Expert 1 holds a PhD in biophysics and is interested in digital pathology to investigate the regulation of the immune system with cancer, infectious, and auto-immune diseases. Expert 2 is an MD PhD studying melanoma progression, evaluating prognostic markers, and seeking to identify novel therapeutic strategies. Only Expert 1 is a co-author of this paper and was part of the requirement analysis.

### Study Setup:

We first introduced the experts to *Cell2Cell* and demonstrated its features. We conducted one-hour-long interviews with both experts and encouraged them to analyze a given dataset in *Cell2Cell* and explain the biological meaning of their findings. Both case studies were conducted over Zoom in a pair analytics setting [7]. While the biomedical experts guided the analysis, we operated the interface. This approach allowed us to identify limitations and improve features for applying *Cell2Cell* to cutting-edge research tasks instead of collecting feedback on user interface details. In the interview, both users gave us feedback on *Cell2Cell* analysis features and data visualization.

### Data:

For our evaluation, we used an immunofluorescence data set, described in Nirmal et al. [35], which contains high-resolution images of cutaneous melanoma samples from 13 patients (11 primary tumors, one locoregional metastasis, and one distant skin metastasis). 70 tissue regions with distinctive morphologies or locations were annotated, averaging 5.5 histological ROIs per specimen. Each image in the data set features 28 plex CyCIF, providing a comprehensive view of the spatial distribution of various cell types and their interactions within the tumor microenvironment. The imaging data, with a lateral resolution of 220 nm, is of considerable size and complexity, as it includes multiple channels representing the expression of different proteins for each cell (28 channels, resolution 1,024 x 1,024 x 55). For the evaluation in one of the CyCIF cubes, almost all cell centers were annotated by hand. The graph itself was then generated as described in Sec. 6.1.

### 10.1 Case Study 1: Verifying Interaction Profiles

To evaluate *Cell2Cell*’s accuracy, our expert aimed to recreate measurements from a previous study on primary cutaneous melanoma [35]. For the original study, the experts used the following steps to create the figure presented in the paper. First, the researchers find channels of interest using an image viewer of choice. They find channel settings (thresholds and value ranges to filter noise from the actual protein signal) and create images for each channel and area of interest. The experts manually extract a rectangular image part between the cells of interest. They use multiple tools to convert the image information present in the area of interest to an intensity profile along the long side of the rectangle. This process has to be carried out for each channel individually. The resulting information is then visualized using standard visualization tools. The data was only gathered at a singular depth section. While the manual workflow was sufficient at the time, it does not scale to many interactions and is difficult to reproduce: The position and size of the rectangle were not based on prior cell center labels or a cell graph, and the approach was restricted to finding and analyzing cells in one section (slice), not supporting oblique 3D orientations.

#### Analysis:

The scientist began by activating the DNA and HLAA channels to visualize the nuclei and then searched for specific markers, namely CD8A and SOX10. Using the right side panel, he adjusted the thresholds for these markers to enhance their visibility. He then used the proofreading features to examine the graph structure and fine-tune cell centers to align with the original experiment’s measurement area [35]. For the interaction profiles, the expert found the cylinder shape and a voxel radius of 25 to recreate the original conditions most closely. He set the thresholds for SOX10 and CD3D to suppress noise in those channels. Finally, he analyzed the interaction profile for the markers (CD3D, CD8A, and SOX10) along his targeted edge and obtained the desired results (Fig. 8 I). Using the interactive functionality to zoom and change viewing angles, Expert 1 was able to identify and extract the contact zone (i.e., immune synapse) between a cytotoxic T lymphocyte and a tumor cell (Fig. 8 I, left) To study the interaction more closely, the expert asked us to switch to the bi-conic shape (see Fig 8 II), which had not been available in the previous analyses. According to our expert, the cone shape represents the area of interest more accurately but is less intuitive when manually measuring the data.

### 10.2 Case Study 2: Interaction of Tumor and T-Cells

In this study, our expert was interested in analyzing the interaction between tumor and t-cells, specifically in the suppressive role of the PDL1-PD1 pathway, preventing tumors from being targeted by t-cells.

#### Analysis:

The expert began by selecting a DNA channel to visualize the dataset’s volumetric properties. Subsequently, we adjusted the brightness and colormap of the DNA channel to enhance the visibility of nuclei. We used the graph editing function to verify and adjust cell center positions. After this proofreading step, the expert activated PDL1 in the volume renderer to identify potential PDL1-PD1 interactions. This revealed one PDL1 positive cell (Fig. 8 IV, a) located in the top center region. In addition, she saw that cell *a* was making contact with a PD1 positive T cell (Fig. 8, b). To ascertain the phenotype of cell *a* and its interacting partner *b*, we activated *Cell2Cell*’s heatmap feature, providing a glance at all expressed proteins along selected interaction edges. After proteins of interest were identified, we switched to the detailed line chart views to measure SOX10 (a tumor marker) and CD3D (immune marker), HLAA (marking cell membrane), along with inhibitory pathway PDL1-PD1. This is depicted in (Fig. 8, III). To visually corroborate this result spatially, the expert also activated SOX10, HLAA, and PDL1 in the volume renderer, which exposed a cluster of SOX10-positive cells surrounding our target cell (Fig. 8, IV). We selected the respective interaction edges and employed *Cell2Cell*’s sorting functionality to rank the edges by similarity. This revealed the PDL1-PD1 expression as the main difference to the immune-cancer cell interaction *e*2, while the signaling between the two cancer cells *c,d* was ranked most different. Based on the SOX10 marker and the nuclear morphology, the domain expert concluded that cells *c* and *d* were tumor cells. We activated the polarization view to gain a cell-centric perspective (Fig. 8, IV). This revealed that cell *a* expressed SOX10 primarily on the side opposite to the PDL1-PD1 interaction. The polarization plot summarizes findings from a context-aware perspective.

### 10.3 Case Study 3: The Role of Macrophages

The third case study investigates T-cell interactions with macrophages. Macrophages are a type of white blood cell that surrounds and kills microorganisms, removes dead cells, and stimulates the action of other immune system cells [6]. However, a hypothesis is that macrophages, because of their capability to suppress inflammation, also provide a supportive environment for tumor growth by suppressing T-cell recruitment. Our second domain expert, Expert 2, previously evaluated where physical contact between cell membranes occurred by visualizing the polarization markers P(PD1, PDL1) in 2D slices. One issue during the initial analysis was that her proximity analysis did not consider whether cells made contact. *Cell2Cell* gives her the opportunity to explore these interactions more efficiently, in 3D, and at scale.

#### Analysis:

**Fig. 9:**
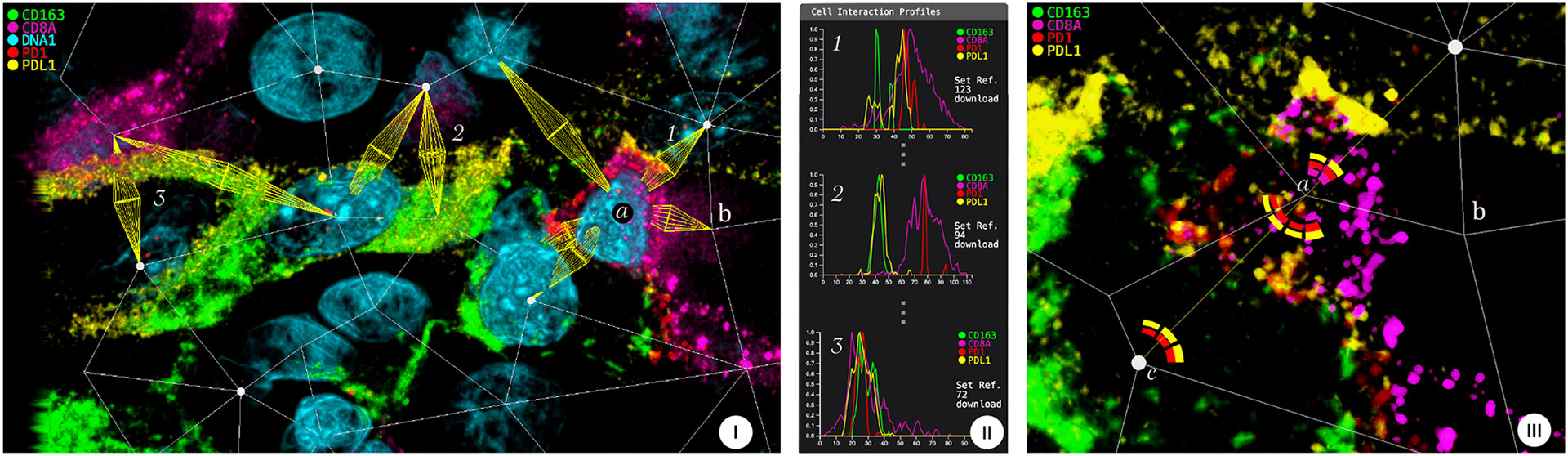
Case Study 3: **(I)** a macrophage-rich area with PD1-PDL1 expressed between macrophages (cells surrounded by CD163 in green) and t-cells (a) (expressing CD8). **(II)** Similarity sorting reveals (1) profiles with the two proteins being expressed in proximity at the cell membranes; (2) profiles with clearly separated PDL1, PD1 expression in the cell centers. (3) Signals are overall shifted towards the t-cell. **(III)** Cell-centric view of (a,c) showing a polarization of PDL1 and PD1 towards each other and CD8 on the opposite side. Cell (b) is negative for available type markers.

We started the analysis by activating channel DNA that stains all cell nuclei. The expert then wanted us to look for specific cancer (SOX10), immune (CD3, CD4, CD8), and macrophage (CD163) markers to quickly spot where in the volume they were expressed. The CD163 channel revealed a macrophage area in the lower left. We marked the area in the volume to select all interaction edges involving macrophages. The expert then asked us to activate the checkpoint pathway markers PDL1/PD1 which appeared between many of the macrophages and t-cells (Fig 9, I). We quickly found one textbook example of such an interaction and sorted the list of interactions by similarity. This revealed that some interactions only expressed PDL1 and PD1 directly together on the cell membranes while others expressed these proteins only within the cells, leaving a gap in between the profile (Fig 9, II). Latter cases could possibly be earlier stages of the pathway formation. It is, however, also likely that the expressions are parts of interactions with other cells outside the data cube. Following the expert, we zoomed in on an interaction where PDL1 and PD1 signals were spatially close to one another. We then activated the polarization view to get a cell-centric understanding of marker distributions. This revealed an interesting case of CD8 expression towards the opposite side of a PDL1-PD1 pathway (Fig 9, III). Moreover, in the neighborhood of this CD8 was a cell that stained negative for the immune, cancer, and macrophage markers. To gain a quick understanding of the cell type involved in the CD8 interaction, we activated the channel heatmap profile. This gave us a quick overview of all involved proteins. Surprisingly, none of the available protein markers in the dataset marked this cell. Changing the camera’s viewport revealed that the call was only half captured by the volume, motivating the need for thicker sectioned data and evaluating additional protein markers in further experiments.

### 10.4 User Feedback

After the analysis sessions, we solicited user feedback regarding its potential to aid their research. Expert 1 found the ability to analyze any interaction on the fly remarkable, as it helps him better understand biological processes. He found the heatmap visualization useful for analyzing all channels at a glance while still having the possibility to switch to more line charts. The expert remarked that our tool will be essential and marks a starting point for future, more elaborate cell-to-cell interaction analysis. Expert 2 highlighted the capability to reveal new protein interactions previously obscured by known interactions, particularly when using the interaction profile visualized as line charts. The expert found value in the efficient combination of volume view and interaction heatmaps and line charts. Expert 2 observed that while there was always room for improvement, the current speed of *Cell2Cell* was notably better than what they experienced in their prior workflow (see Sec. 10.1). The polarization visualization stood out as particularly beneficial for analyzing interactions within tumor cell clusters.

Both experts expressed interest in the ability to import cell types from a list into the tool for validation and comparison with the interactions, which we subsequently implemented. Expert 2 suggested a possible extension to this feature, which could involve implementing a semi-automated cell typing algorithm, allowing domain experts to import a set of rules that translate interactions directly into cell types based on specific marker measurements. Expert 2 was interested in the prospects of handling larger, forthcoming datasets. *Cell2Cell* is scalable and will accommodate a moderate increase in volume sizes. If scalability becomes a concern, the domain expert suggested that cropping images to regions of interest would serve as an acceptable workaround.

In summary, both domain experts affirmed that our tool would benefit their research and that they trust the results it produces. They highlighted the value of our interaction comparison and polarization visualizations in providing new insights into the data.

## 11 Limitations and Future Work

While our multichannel volume rendering approach offers significant benefits for analyzing cellular interactions in 3D data, it is important to recognize its limitations and avenues for future work.

### Cell Segmentation and Image Quality.

Our method relies on accurate 3-D coordinates for cell centroids and manual refinement. The spherical approximation of cells as interacting spheres (approx. 1.5-2 nuclear radii) is scalable but ignores interactions at a distance mediated by projections > 4 nuclear radii [35]. Cell boundary segmentation could provide a more accurate shape representation. Besides segmentation, image quality also affects interaction analysis. The signal-to-noise ratio in the image channels has to be high enough for an accurate analysis.

### Multi-channel Volume Rendering.

Our current implementation renders six channels simultaneously. This limitation maintains clarity and avoids overplotting and mixing of too many colors. Advances in transfer function design and renderings could enable simultaneous visualization of more channels while preserving clarity and interpretability. To make the rendering scalable to larger data, "Residency Octree" [22] presents a novel web-based technique [22], combining the advantages of out-of-core volume rendering using page tables with those of standard octrees. We aim to integrate this technique in the near future.

### Complex Interaction Modeling.

*Cell2Cell* reduces localized 3D distributions to one dimension (interaction and polarization profiles) to quantify the signal. While this works well to capture the most significant information, deep learning could be used to learn more complex features from segmented data. Autoencoders could compress the multiplex data to a lower dimensional representation, and graph neural networks could be utilized to learn interactions of larger cellular communities and for improved ranking and clustering [49]. Visual analytics could help to steer such algorithms interactively and explain results.

### Imaging Modalities.

We showed the utility of our approach using CyCIF datasets provided by our collaborators. These datasets have a high resolution and feature multiple channels presenting various biological meaningful structures to investigate. In the next step, we want to enable scientists working with other imaging techniques like CODEX or Image Mass Cytometry to also use *Cell2Cell* for the analysis of their data. Access to 3D data on a single cell level featuring multiple channels is limited and therefore we rely on the advancement of imaging techniques to deliver more data for the analysis in *Cell2Cell.*

## 12 Conclusions

We have presented *Cell2Cell*, which combines a two-pronged approach for analyzing cell-to-cell interactions in 3D multi-channel volume data. Our tool is designed to aid domain experts in advancing their understanding of the intricate mechanisms underlying cellular interactions in the context of cancer and immunology research. First, our tool computes a cell graph where cell centers are represented as nodes, and edges depict interactions between cells. We then automatically analyze and visualize marker intensities along these edges, enabling the creation of interaction profiles and facilitating comparative analysis using both spatial and abstract data views. Second, our cell-centered approach allows scientists to visually analyze 3D cell polarization based on marker distributions, providing an orthogonal perspective on cell interactions. While such analysis has been carried out manually on an individual cell-to-cell basis, our computational approach enables analysis at scale. Incorporating these analytics into a web-based volume rendering interface enables effective and efficient visual exploration and verification of the computed measures. This "in-situ" visual exploration (close to the actual imaging data) is essential, empowering pathologists to leverage their deep visual understanding of healthy and diseased tissue features.

## Supplementary Material

supp1-3456406

## Figures and Tables

**Fig. 4:**
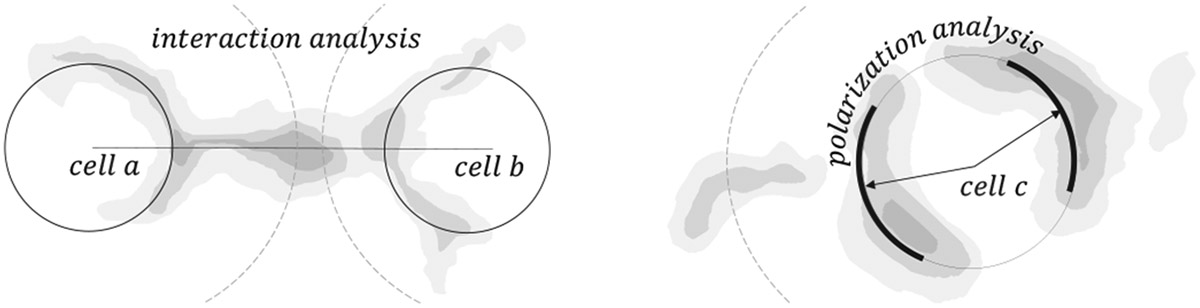
Simplified illustration of goals G2 and G3. Left: Interaction Analysis (G2) focuses on analyzing protein expressions between a pair of cells (*a* and *b*). Right: Polarization Analysis (G3) focuses on a cell-centric understanding of the polar distribution of protein expressions around a cell (*c*). The inner ring illustrates the nuclei, and the outer dashed line illustrates the membrane.

## References

[1] Allen Institute for Cell Science (2018). hiPSC Single-cell Image Dataset [dataset]. Available from: allencell.org/3d-cell-viewer, Last accessed: 04/01/2023.

[2] Imaris for Cell Biologists - Imaris. Available from: imaris.oxinst.com/products/imaris-for-cell-biologists, Last accessed: 08/04/2024.

[3] NumPy - The fundamental package for scientific computing with Python. Available from: numpy.org, Last accessed: 06/29/2024.

[4] Three.js – JavaScript 3D Library. Available from: threejs.org, Last accessed: 03/28/2024.

[5] Welcome to Flask — Flask Documentation (2.3.x). Available from: flask.palletsprojects.com, Last accessed: 06/29/2024.

[6] Definition of macrophage - NCI Dictionary of Cancer Terms - NCI, Feb. 2011. Available from: www.cancer.gov/publications/dictionaries/cancer-terms/def/macrophage, Last Accessed: 03/29/2024.

[7] Arias-Hernandez R, Kaastra LT, Green TM, and Fisher B. Pair Analytics: Capturing Reasoning Processes in Collaborative Visual Analytics. In 2011 44th Hawaii International Conference on System Sciences, pp. 1–10, Jan. 2011. ISSN: 1530-1605. doi: 10.1109/HICSS.2011.339

[8] Berg S, Kutra D, Kroeger T, Straehle CN, Kausler BX, Haubold C, Schiegg M, Ales J, Beier T, Rudy M, Eren K, Cervantes JI, Xu B, Beuttenmueller F, Wolny A, Zhang C, Koethe U, Hamprecht FA, and Kreshuk A. ilastik: interactive machine learning for (bio)image analysis. Nature Methods, 16(12):1226–1232, Dec. 2019. Number: 12 Publisher: Nature Publishing Group. doi: 10.1038/s41592-019-0582-9 31570887

[9] Berndt DJ and Clifford J. Using dynamic time warping to find patterns in time series. In Proceedings of the 3rd International Conference on Knowledge Discovery and Data Mining, AAAIWS’94, pp. 359–370. AAAI Press, Seattle, WA, July 1994. doi: 10.5555/3000850.3000887

[10] Bostock M, Ogievetsky V, and Heer J. D^3^ Data-Driven Documents. IEEE Transactions on Visualization and Computer Graphics, 17(12):2301–2309, Dec. 2011. doi: 10.1109/TVCG.2011.185 22034350

[11] Chasman D, Fotuhi Siahpirani A, and Roy S. Network-based approaches for analysis of complex biological systems. Current Opinion in Biotechnology, 39:157–166, June 2016. doi: 10.1016/j.copbio.2016.04.007 27115495

[12] Chiu C-L, Clack N, and The-Napari-Community. napari: a Python Multi-Dimensional Image Viewer Platform for the Research Community. Microscopy and Microanalysis, 28(S1):1576–1577, Aug. 2022. Publisher: Cambridge University Press. doi: 10.1017/S1431927622006328

[13] Ding K, Zhou M, Wang Z, Liu Q, Arnold CW, Zhang S, and Metaxas DN. Graph Convolutional Networks for Multi-modality Medical Imaging: Methods, Architectures, and Clinical Applications. arXiv preprint, Feb. 2022. arXiv preprint. doi: 10.48550/arXiv.2202.08916

[14] Draper GM, Livnat Y, and Riesenfeld RF. A Survey of Radial Methods for Information Visualization. IEEE Transactions on Visualization and Computer Graphics, 15(5):759–776, Sept. 2009. doi: 10.1109/TVCG.2009.23 19590103

[15] Drees D, Leistikow S, Jiang X, and Linsen L. Voreen – An Open-source Framework for Interactive Visualization and Processing of Large Volume Data, July 2022. arXiv:2207.12746 [cs]. doi: 10.48550/arXiv.2207.12746

[16] Fritz L, Hadwiger M, Geier G, Pittino G, and Gröller ME. A Visual Approach to Efficient Analysis and Quantification of Ductile Iron and Reinforced Sprayed Concrete. IEEE Transactions on Visualization and Computer Graphics, 15(6):1343–1350, Nov. 2009. doi: 10.1109/TVCG.2009.115 19834207

[17] Gabriel KR and Sokal RR. A New Statistical Approach to Geographic Variation Analysis. Systematic Biology, 18(3):259–278, Sept. 1969. doi: 10.2307/2412323

[18] Gehlenborg N, O’Donoghue SI, Baliga NS, Goesmann A, Hibbs MA, Kitano H, Kohlbacher O, Neuweger H, Schneider R, Tenenbaum D, and Gavin A-C. Visualization of omics data for systems biology. Nature Methods, 7(3):S56–S68, Mar. 2010. Number: 3 Publisher: Nature Publishing Group. doi: 10.1038/nmeth.1436 20195258

[19] Gleicher M, Albers D, Walker R, Jusufi I, Hansen CD, and Roberts JC. Visual comparison for information visualization. Information Visualization, 10(4):289–309, Oct. 2011. Publisher: SAGE Publications. doi: 10.1177/1473871611416549

[20] C. Z. M. S. C. R. GmbH. ZEISS arivis - The Scientific Image Analysis Platform.

[21] Grundy E, Jones MW, Laramee RS, Wilson RP, and Shepard EL, Visualisation of Sensor Data from Animal Movement. Computer Graphics Forum, 28(3):815–822, 2009. doi: 10.1111/j.1467-8659.2009.01469.x

[22] Herzberger L, Hadwiger M, Krüger R, Sorger P, Pfister H, Gröller E, and Beyer J. Residency Octree: A Hybrid Approach for Scalable Web-Based Multi-Volume Rendering. IEEE Transactions on Visualization and Computer Graphics, 30(1):1380–1390, Jan. 2024. doi: 10.1109/TVCG.2023.3327193 37889813 PMC10840607

[23] Javed W, McDonnel B, and Elmqvist N. Graphical Perception of Multiple Time Series. IEEE Transactions on Visualization and Computer Graphics, 16(6):927–934, Nov. 2010. Conference Name: IEEE Transactions on Visualization and Computer Graphics. doi: 10.1109/TVCG.2010.162 20975129

[24] Jessup J, Krueger R, Warchol S, Hoffer J, Muhlich J, Ritch CC, Gaglia G, Coy S, Chen Y-A, Lin J-R, Santagata S, Sorger PK, and Pfister H. Scope2Screen: Focus+Context Techniques for Pathology Tumor Assessment in Multivariate Image Data. IEEE Transactions on Visualization and Computer Graphics, 28(1):259–269, Jan. 2022. doi: 10.1109/TVCG.2021.3114786 34606456 PMC8805697

[25] Johnson GR, Donovan-Maiye RM, and Maleckar MM. Building a 3D Integrated Cell. Technical report, bioRxiv, Dec. 2017. Section: New Results Type: article. doi: 10.1101/238378

[26] Kantorovich LV. Mathematical Methods of Organizing and Planning Production. Management Science, 6(4):366–422, July 1960. Publisher: INFORMS. doi: 10.1287/mnsc.6.4.366

[27] Karatas OH and Toy E. Three-dimensional imaging techniques: A literature review. European Journal of Dentistry, 8(1):132, Mar. 2014. Publisher: Dental Investigations Society. doi: 10.4103/1305-7456.126269 24966761 PMC4054026

[28] Kazmi IK, You L, and Zhang JJ. A Survey of 2D and 3D Shape Descriptors. In Imaging and Visualization 2013 10th International Conference Computer Graphics, pp. 1–10, Aug. 2013. doi: 10.1109/CGIV.2013.11

[29] Krueger R, Han Q, Ivanov N, Mahtal S, Thom D, Pfister H, and Ertl T. Bird’s-Eye - Large-Scale Visual Analytics of City Dynamics using Social Location Data. Computer Graphics Forum, 38(3):595–607, 2019. doi: 10.1111/cgf.13713

[30] Lin J-R, Izar B, Wang S, Yapp C, Mei S, Shah PM, Santagata S, and Sorger PK. Highly multiplexed immunofluorescence imaging of human tissues and tumors using t-CyCIF and conventional optical microscopes. eLife, 7:e31657, July 2018. doi: 10.7554/eLife.31657 29993362 PMC6075866

[31] Lin J-R, Wang S, Coy S, Chen Y-A, Yapp C, Tyler M, Nariya MK, Heiser CN, Lau KS, Santagata S, and Sorger PK. Multiplexed 3D atlas of state transitions and immune interaction in colorectal cancer. Cell, 186(2):363–381.e19, Jan. 2023. doi: 10.1016/j.cell.2022.12.028 36669472 PMC10019067

[32] Manz T, Gold I, Patterson NH, McCallum C, Keller MS, Herr BW, Börner K, Spraggins JM, and Gehlenborg N. Viv: multiscale visualization of high-resolution multiplexed bioimaging data on the web. Nature Methods, 19(5):515–516, May 2022. Number: 5 Publisher: Nature Publishing Group. doi: 10.1038/s41592-022-01482-7 35545714 PMC9637380

[33] Marin-Acevedo JA, Dholaria B, Soyano AE, Knutson KL, Chumsri S, and Lou Y. Next generation of immune checkpoint therapy in cancer: new developments and challenges. Journal of Hematology & Oncology, 11:39, Mar. 2018. doi: 10.1186/s13045-018-0582-8 29544515 PMC5856308

[34] Mörth E, Haldorsen IS, Bruckner S, and Smit NN. Paraglyder: Probe-driven interactive visual analysis for multiparametric medical imaging data. In Advances in Computer Graphics: 37th Computer Graphics International Conference, 13 pages, p. 351–363. Springer-Verlag, Berlin, Heidelberg, 2020. doi: 10.1007/978-3-030-61864-3_29

[35] Nirmal AJ, Maliga Z, Vallius T, Quattrochi B, Chen AA, Jacobson CA, Pelletier RJ, Yapp C, Arias-Camison R, Chen Y-A, Lian CG, Murphy GF, Santagata S, and Sorger PK. The Spatial Landscape of Progression and Immunoediting in Primary Melanoma at Single-Cell Resolution. Cancer Discovery, 12(6):1518–1541, June 2022. doi: 10.1158/2159-8290.CD-21-1357 35404441 PMC9167783

[36] Nobre C, Meyer M, Streit M, and Lex A. The State of the Art in Visualizing Multivariate Networks. Computer Graphics Forum, 38(3):807–832, 2019. doi: 10.1111/cgf.13728

[37] Pavlopoulos GA, Secrier M, Moschopoulos CN, Soldatos TG, Kossida S, Aerts J, Schneider R, and Bagos PG. Using graph theory to analyze biological networks. BioData Mining, 4(1):10, Dec. 2011. doi: 10.1186/1756-0381-4-10 21527005 PMC3101653

[38] Peyré G and Cuturi M. Computational Optimal Transport: With Applications to Data Science, Foundations and Trends in Machine Learning, 11(5-6):355–607, 2019. doi: 0.1561/2200000073

[39] Richards TA, Massana R, Pagliara S, and Hall N. Single cell ecology. Philosophical Transactions of the Royal Society B: Biological Sciences, 374(1786):20190076, Oct. 2019. Publisher: Royal Society. doi: 10.1098/rstb.2019.0076 PMC6792453 31587644

[40] Schapiro D, Jackson HW, Raghuraman S, Fischer JR, Zanotelli VRT, Schulz D, Giesen C, Catena R, Varga Z, and Bodenmiller B. histoCAT: analysis of cell phenotypes and interactions in multiplex image cytometry data. Nature Methods, 14(9):873–876, Sept. 2017. Number: 9 Publisher: Nature Publishing Group. doi: 10.1038/nmeth.4391 28783155 PMC5617107

[41] Schapiro D, Sokolov A, Yapp C, Chen Y-A, Muhlich JL, Hess J, Creason AL, Nirmal AJ, Baker GJ, Nariya MK, Lin J-R, Maliga Z, Jacobson CA, Hodgman MW, Ruokonen J, Farhi SL, Abbondanza D, McKinley ET, Persson D, Betts C, Sivagnanam S, Regev A, Goecks J, Coffey RJ, Coussens LM, Santagata S, and Sorger PK. MCMICRO: a scalable, modular image-processing pipeline for multiplexed tissue imaging. Nature Methods, 19(3):311–315, Mar. 2022. Number: 3 Publisher: Nature Publishing Group. doi: 10.1038/s41592-021-01308-y 34824477 PMC8916956

[42] Sedlmair M, Meyer M, and Munzner T. Design Study Methodology: Reflections from the Trenches and the Stacks. IEEE Transactions on Visualization and Computer Graphics, 18(12):2431–2440, Dec. 2012. doi: 10.1109/TVCG.2012.213 26357151

[43] Somarakis A, Van Unen V, Koning F, Lelieveldt B, and Höllt T. Ima-CytE: Visual Exploration of Cellular Micro-Environments for Imaging Mass Cytometry Data. IEEE Transactions on Visualization and Computer Graphics, 27(1):98–110, Jan. 2021. doi: 10.1109/TVCG.2019.2931299 31369380

[44] Ternes L, Dane M, Gross S, Labrie M, Mills G, Gray J, Heiser L, and Chang YH. ME-VAE: Multi-Encoder Variational AutoEncoder for Controlling Multiple Transformational Features in Single Cell Image Analysis. Technical report, bioRxiv, May 2021. Section: New Results Type: article. doi: 10.1101/2021.04.22.441005

[45] Troidl J, Cali C, Gröller E, Pfister H, Hadwiger M, and Beyer J. Barrio: Customizable Spatial Neighborhood Analysis and Comparison for Nanoscale Brain Structures. Computer Graphics Forum, 41(3):183–194, 2022. doi: 10.1111/cgf.14532

[46] Van Der Walt S, Schönberger JL, Nunez-Iglesias J, Boulogne F, Warner JD, Yager N, Gouillart E, and Yu T. scikit-image: image processing in Python. PeerJ, 2:e453, June 2014. doi: 10.7717/peerj.453 25024921 PMC4081273

[47] Warchol S, Krueger R, Nirmal AJ, Gaglia G, Jessup J, Ritch CC, Hoffer J, Muhlich J, Burger ML, Jacks T, Santagata S, Sorger PK, and Pfister H. Visinity: Visual Spatial Neighborhood Analysis for Multiplexed Tissue Imaging Data. IEEE Transactions on Visualization and Computer Graphics, pp. 1–11, 2022. doi: 10.1109/TVCG.2022.3209378 PMC10043053 36170403

[48] Wilson RP, Williams HJ, Holton MD, di Virgilio A, Börger L, Potts JR, Gunner R, Arkwright A, Fahlman A, Bennett NC, Alagaili A, Cole NC, Duarte CM, and Scantlebury DM. An “orientation sphere” visualization for examining animal head movements. Ecology and Evolution, 10(10):4291–4302, 2020. doi: 10.1002/ece3.6197 32489597 PMC7246194

[49] Yang F, Fan K, Song D, and Lin H. Graph-based prediction of Protein-protein interactions with attributed signed graph embedding. BMC Bioinformatics, 21(1):323, Dec. 2020. doi: 10.1186/s12859-020-03646-8 32693790 PMC7372763

[50] Yi JS, Kang Y. a., Stasko J, and Jacko J. Toward a Deeper Understanding of the Role of Interaction in Information Visualization. IEEE Transactions on Visualization and Computer Graphics, 13(6):1224–1231, Nov. 2007. doi: 10.1109/TVCG.2007.70515 17968068

[51] Zeng W, Fu C-W, Müller Arisona S, Schubiger S, Burkhard R, and Ma K-L. Visualizing the Relationship Between Human Mobility and Points of Interest. IEEE Transactions on Intelligent Transportation Systems, 18(8):2271–2284, Aug. 2017. doi: 10.1109/TITS.2016.2639320

